# A Closed-Loop Audit for Orthopedic Trauma Operation Notes Comparing Typed Electronic Notes With Handwritten Notes

**DOI:** 10.7759/cureus.26808

**Published:** 2022-07-13

**Authors:** Fitzgerald Anazor, Vusumuzi Sibanda, Aisha Abubakar, Mutmainah Ekungba-Adewole, Hany Elbardesy, Baljinder S Dhinsa

**Affiliations:** 1 Trauma and Orthopaedics, William Harvey Hospital, Ashford, GBR

**Keywords:** trauma and orthopedic surgery, quality improvement, audit and feedback, medical records, operation notes

## Abstract

Introduction

Operation notes are important documents for ensuring patient safety, effective communication between clinicians, and for medicolegal purposes. It is essential that they are clear and accurate. We audited the quality of our operation notes against the Royal College of Surgeons (RCS) of England's Good Surgical Practice Guidelines.

Methods

This was a prospective audit of 99 orthopedic trauma operation notes. In the first cycle, we audited 58 operation notes for orthopedic trauma surgical procedures. We audited 17 parameters per note. We presented our findings, implemented changes including the use of a typed operation note template, and performed a re-audit using 41 operation notes.

Results

Our documentation for 3/17 parameters was up to standard in both cycles. Post-intervention, there was an improvement in documentation for 12/17 of the parameters with marked improvements in indication for surgery (45% vs 75%), tourniquet time (20% vs 45%), antibiotic prophylaxis (71% vs 89%), closure technique (62% vs 86%) and detailed postoperative instruction (40% vs 92%). Other parameters, particularly estimated blood loss (7% vs 8%) remained unchanged. In the second cycle, we noted that 25% of the typed notes had 100% compliance with the standards, whereas no handwritten note achieved this. However, there was no statistically significant difference in the mean number of correctly documented parameters between the typed and handwritten notes (p < 0.05).

Conclusion

The use of operation note templates (preferably typed) can improve appropriate documentation in orthopedic trauma operation notes. These templates should be made easily accessible to all surgeons. We will recommend orthopedic trauma units to apply similar non-rigid templates that can be tailored to suit different categories of trauma surgery.

## Introduction

An operation note is an important medicolegal document that clearly “tells the story” of the intra-operative surgical events from the surgeon’s perspective. It also provides useful information for the postoperative care of patients on the ward and on discharge. On the other hand, it does not include the full details of other anesthetic and perioperative events. A concise electronic patient record that contains these documents in an easily accessible and legible format will be of immense benefit to clinicians and also improve patient safety. A good operation note will also provide easily accessible information for academic and educational reasons [1]. Documentation is an important form of written communication needed for the continuation of care, and patients may come to harm if this is not done properly.

We carried out this prospective closed-loop audit because we noticed that many operation notes were missing key parameters like estimated blood loss, intra-operative findings, tourniquet time, indication for surgery, intra-operative findings, and a clear postoperative plan. The lack of a clear and legible postoperative and venous thromboprophylaxis plan led to junior doctors complaining on multiple occasions about challenges with completing electronic discharge notes (EDNs) and nurses struggling to comprehend the documented postoperative plan on several occasions. This, we felt, was a possible risk to patient safety.

Current practice by most surgeons in our institution is to document either on handwritten notes or on a typed electronic proforma. Despite these, there appeared to be a lack of awareness about what parameters should be included. Some handwritten notes were deemed adequate in content but demonstrated poor legibility.

A typical orthopedic trauma list can place pressure on surgeons in terms of patient prioritization and complex multidisciplinary working and on patients with complex medical comorbidities [2,3]. This means that the importance of an easily accessible and user-friendly operation note template cannot be over-emphasized in order to allow surgeons to complete these notes in between operating cases so that the continuation of patient care will not be affected in the post-anesthesia recovery room or in the wards.

Although there is a problem of multiple documentation where some of the perioperative parameters that are typically found in operation notes might also be documented in other theater paper-based or electronic systems, it is the surgeon’s utmost responsibility to ensure that a good operation note is completed and to avoid assuming that some of the undocumented parameters will be found in these alternative systems. However, synchronizing perioperative patient care documents on a single electronic patient record will help reduce this problem.

This six-month closed-loop audit was carried out with special emphasis on emergency and planned trauma cases performed in the orthopedic trauma unit at the William Harvey Hospital, a relatively busy district general hospital in the southeast of England within the East Kent Hospitals University NHS Foundation Trust. Our goal was to achieve 100% compliance with all the audited parameters on completion of the audit loop. We also compared the handwritten and typed operation notes for the second cycle cohort in our audit to ascertain if there were significant differences between these methods of documentation. We only performed this subgroup analysis following the implementation of the recommendations after the first cycle.

## Materials and methods

This study was registered with the hospital trust’s clinical audit team as an audit project with the registration number SA/19/22-23. Approval was obtained before commencing data collection. All data were collected anonymously, and there was no change to direct patient care pathways.

The audit standard employed was the Royal College of Surgeons (RCS) of England's Good Surgical Practice Guidelines on operation notes (2014) section 1.3 which states: “Record your work clearly, accurately and legibly.” “Surgeons must ensure that accurate, comprehensive, legible and contemporaneous records are maintained of all their interactions with patients.” “Ensure that there are clear (preferably typed) operative notes for every procedure. The notes should accompany the patient into recovery and to the ward and should give sufficient detail to enable continuity of care by another doctor” [4]. Eighteen parameters were mentioned in the RCS guidelines. We excluded “any extra procedures” and “problems encountered” in order to simplify data collection and reduce variation. We also included tourniquet time where applicable as our audit was focused on orthopedic trauma cases (see Table 1). These standards are also in compliance with the General Medical Council (GMC) Guidance (Chapter 6) on keeping medical records [5].

**Table 1:** Modified from the Royal College of Surgeons Good Surgical Practice 2014 operation note documentation guidance. Note that we added “tourniquet time” and “anticipated follow-up plan" to suit orthopedic trauma surgery.

Audit Standard Parameters
Date of surgery
Name of the operating surgeon(s) and assistant(s)
Name of the theater anesthetist
Operative procedure
Indication for the procedure/Operative diagnosis
Intra-operative findings
Incision/approach
Details of specimen/samples taken
Identification of prosthesis/implant
Closure technique
Estimated blood loss
Tourniquet time
Antibiotic prophylaxis
Venous thromboembolism prophylaxis
Detailed immediate postoperative instructions
Anticipated follow-up plan
Signature

A total of 99 orthopedic trauma operation notes were analyzed over both cycles. Clearly identified inclusion and exclusion criteria were set prior to the commencement of data collection for each operation note (see Table 2). Fifty-eight consecutive orthopedic trauma operation notes were analyzed over a 14-day block for the first cycle (November 15-28, 2021), whereas 41 operation notes were analyzed in the second cycle over another 14-day block (April 24-May 7, 2022). Consensus agreement on what each audit parameter meant was agreed upon a priori. Data were collected prospectively; accuracy and validity were verified by at least the lead author and one other member of the audit project team for errors/missing data. Data were uploaded onto the Microsoft excel sheet 2017 version (Microsoft Corporation, Redmond, Washington).

**Table 2:** Inclusion and exclusion criteria guiding data collection for the audited operation notes

Inclusion Criteria	Exclusion Criteria
All orthopedic trauma operation notes for the William Harvey Hospital site performed in the trauma theater	Elective orthopedic cases or operations performed at the other sites within the trust
Handwritten or electronic operation notes	Minor cases done in the emergency department where there was no operation note
All adult and pediatric cases	Injection list cases done in the trauma theater or cases where only a closed manipulation under anesthesia was performed

**Figure 1:**
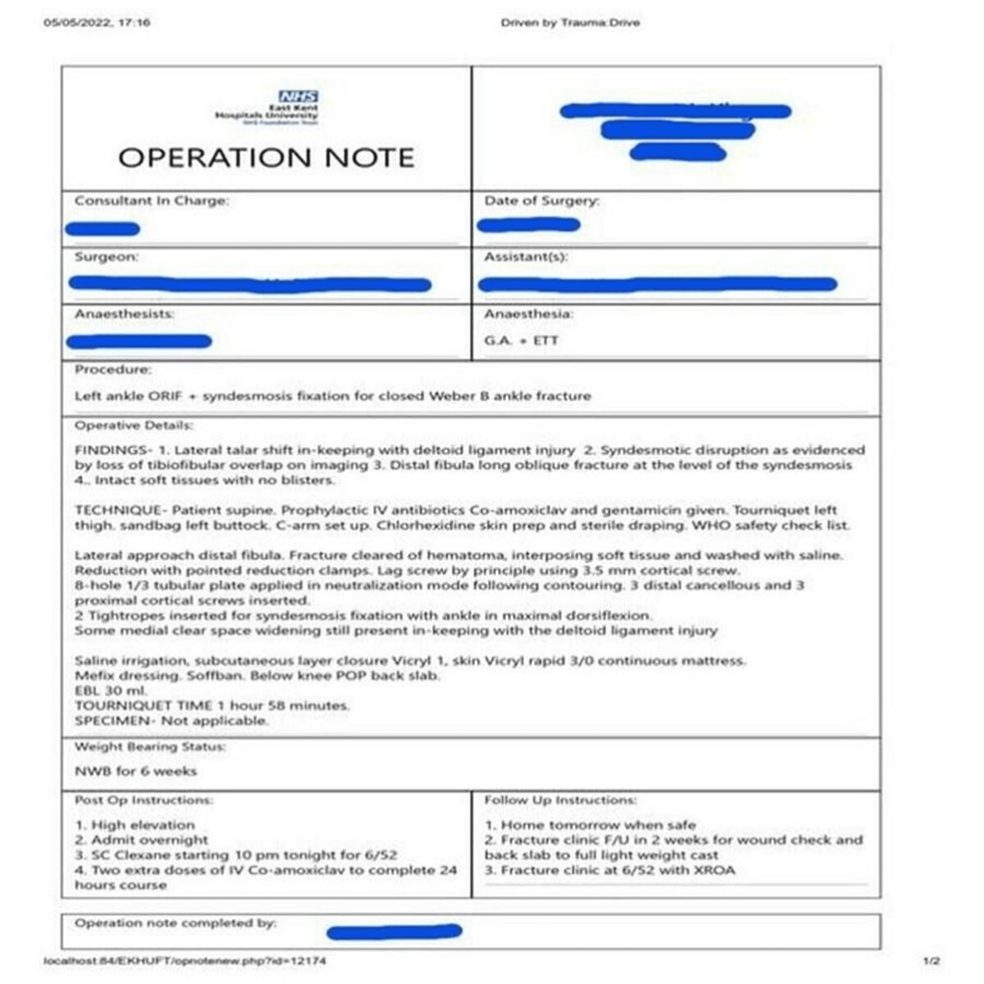
Typed operation note template in our center with documented anonymized details for a completed surgical operation

Findings and recommendations for each audit cycle were presented at the trauma and orthopedic clinical governance meetings in the hospital both in December 2021 and May 2022. Surgeons were educated on the use of the typed proforma (see Figure 1) via the use of live demonstrations, robust discussions, and recorded videos on the importance of these parameters and on how to readily access the operation note templates on the electronic trauma drive system. These completed operation notes were also uploaded immediately to the electronic patient record (EPR) system for easy access in postoperative follow-up clinics.

## Results

**Figure 2:**
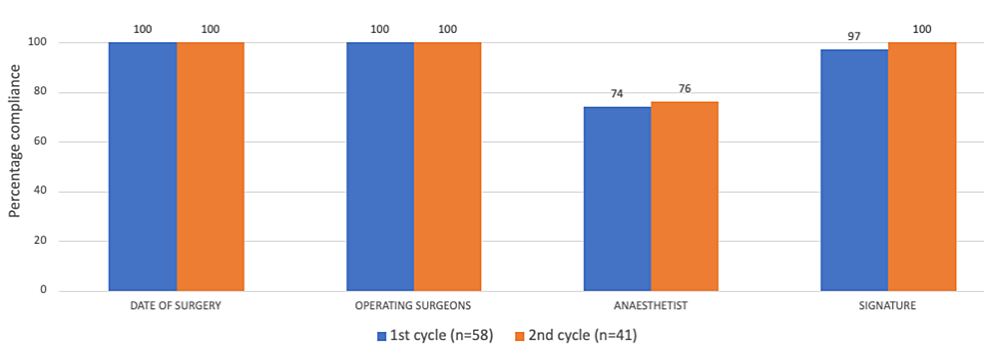
Chart comparing the results of both cycles for correct documentation of the date of surgery and the details of the surgical/anesthesia teams

**Figure 3:**
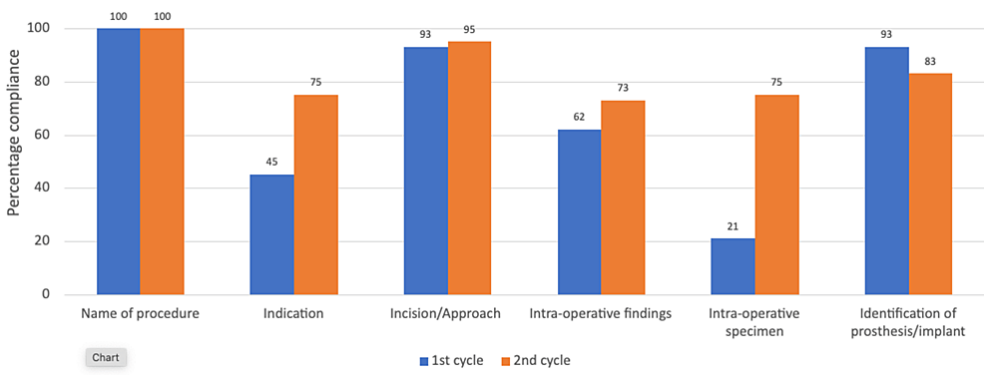
Chart comparing the results of both cycles for correct documentation of the key intra-operative surgical details

**Figure 4:**
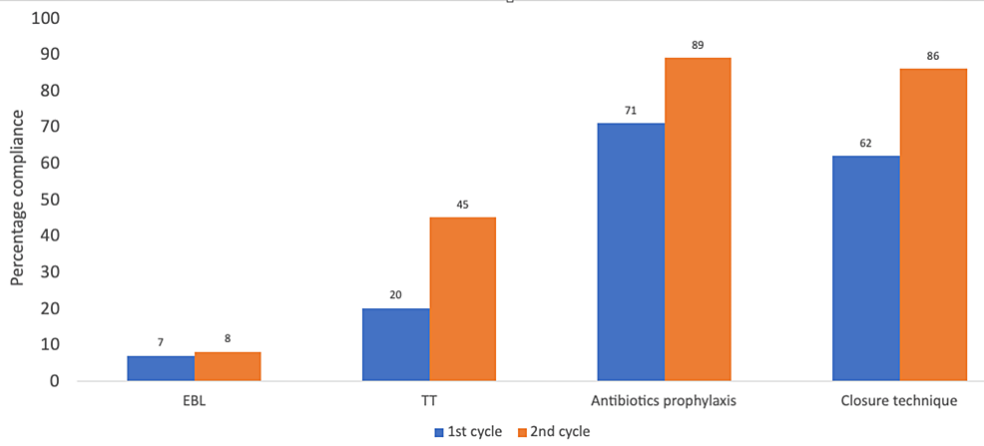
Chart comparing the results of both cycles for correct documentation as applicable for EBL, tourniquet time, antibiotics prophylaxis regimen, and detailed closure technique EBL: Estimated blood loss; TT: Tourniquet time.

**Figure 5:**
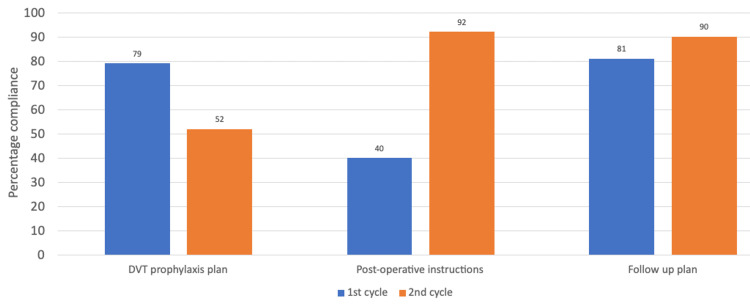
Chart comparing the results of both cycles for correct documentation of a clear deep venous thromboprophylaxis, postoperative care, and follow-up plan DVT: Deep venous thrombosis.

Overall, following the implementation of the interventional activities and recommendations, a comparison of the second cycle to the first cycle revealed marked improvements for 12/17 parameters analyzed (see Figures 2-5). There was more than 70% compliance for 14 out of the 17 parameters on completion of the second cycle compared to 10/17 parameters of the first cycle. No change was noted for three parameters as these were already at 100% compliance (date of surgery, names of the operating surgeons, and name of the procedure).

 

Minimal improvement was found for estimated blood loss (EBL); regression in performance was found for deep venous thrombosis (DVT) prophylaxis plan. Less than 50% compliance for documentation of tourniquet time and estimated/anticipated blood loss persisted in the second cycle. Tourniquet times are also usually documented by the theater nurses/practitioners in the perioperative documents (though this is not always guaranteed). Further analysis was performed for the audited notes in the second cycle following the implementation of the recommendations.

**Figure 6:**
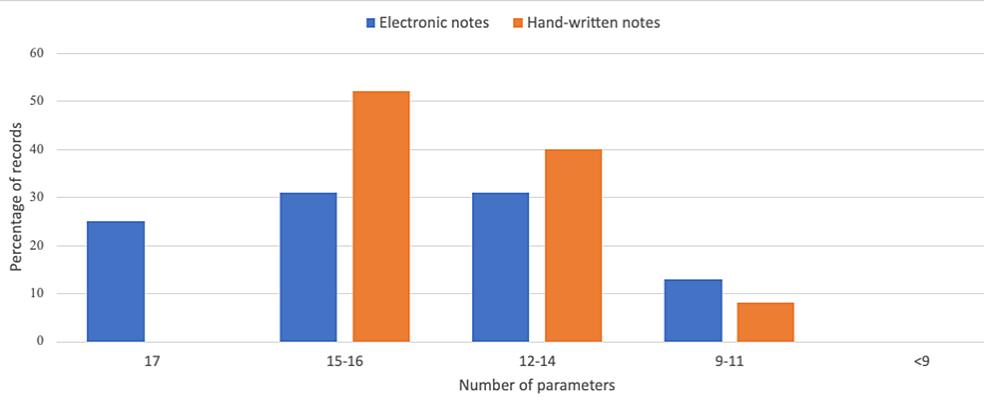
Chart comparing the percentage of compliant operation note records to the number of audited parameters for typed/electronic versus handwritten notes. The maximum number of audited parameters was 17.

We compared the typed operation notes (total number, n = 16) with the handwritten notes (total number, n = 25) in terms of compliance to each of the 17 audited parameters (Figure 6). There was 100% compliance for 25% of the typed notes compared with none of the handwritten notes achieving 100% compliance with all 17 audited parameters. However, for both groups overall, the mean number of correctly documented parameters was 14.375 (SD 2.472) and 14.080 (SD 1.742) for the typed and handwritten notes, respectively. Statistical testing using the unpaired T-test (p = 0.449) at an alpha level of 0.05 showed no statistically significant difference between the two groups.

## Discussion

Electronic notes, when uploaded immediately on the EPR, improve access to operation note records for follow-up clinics. Our audit results show that more typed notes achieved 100% compliance. However, there was no statistically significant difference in terms of the mean number of correctly documented parameters when compared to handwritten notes. A closed-loop audit study by Barritt et al. (2010) for hip hemiarthroplasty operation note records showed an improvement in compliance from 58.7% for handwritten notes to 92.8% when typed electronic notes were introduced [6]. Use of typed, electronic operation notes also eliminates the problem of handwriting legibility typically found in some handwritten operation notes [7,8].

Our closed-loop audit led to improvements in the documentation for 11 out of the 17 parameters with 70% compliance in 14 parameters for the second cycle. However, 100% compliance was noted in only four out of the 17 parameters (name of the procedure, name of operating surgeons, date of surgery, and signature of the surgeon completing the note). A similar audit by Sweed et al. (2014) found 100% compliance only for the name of the surgeon, type of surgery, and date of surgery [9]. These results can be explained by the fact that surgeons will naturally remember to always document their name and type of surgery when completing notes. Most operation note templates usually have pre-populated patient details for electronic notes once the patient hospital identity number is entered into the system or patient stickers are attached for handwritten notes.

We found relatively low compliance for tourniquet time documentation (45%) in the operation notes completed by surgeons. This is comparable to the closed-loop audit by Sweed et al. who found 32% compliance for tourniquet time [9]. This could be explained by the fact that most surgeons assume this will be computed by the theater practitioners as part of the theater audit trail. On the other hand, an audit of elective arthroplasty surgical notes by the Severn Audit and Research Collaborative showed an 83% compliance for tourniquet time documentation [10]. The pressures associated with orthopedic trauma lists compared to elective lists could be one of the reasons for this difference. Nevertheless, we feel it is the surgeon's responsibility to check and document the tourniquet pressure and duration in the operation notes especially for medicolegal and patient safety reasons.

Compliance with documentation of EBL remained low following the completion of this audit loop. We believe this might be due to the perception by some surgeons on the importance of documenting this as some felt the literature evidence to support this is mixed [11,12]. Blood loss estimation in our setting is mainly via visual estimation, weighing of blood-soaked sponges, and measurement of blood lost into suction drains. A systematic review and meta-analysis by Tran et al. (2020) found that visual estimation under-estimated blood loss in 12 out of 13 studies assessed compared to formula methods for calculating EBL [13]. We believe that irrespective of what method is used for checking intra-operative blood loss, it should be properly documented in the operation notes so that in correlation with the patient’s hemoglobin level and physiological parameters, intra-operative blood loss and transfusion requirements can be determined. This is especially important for major orthopedic trauma surgery like hip/femoral diaphyseal fracture surgery and revision surgeries for periprosthetic fractures.

We achieved 92% compliance for documentation of a clear/detailed postoperative instruction and 90% for a clear follow-up plan following completion of our second cycle (from an initial 40% and 81% compliance, respectively, from the first cycle). An audit of hip arthroplasty operations by Menakaya et al. (2013) led to 97% compliance with postoperative instructions [14]. These were the two areas (in addition to a clear venous thromboembolism [VTE] prophylaxis plan) that we felt impacted significantly on patient safety as junior doctors and nurses on the wards identified these as areas they struggled with. A clear postoperative plan for orthopedic trauma surgical operations should ideally include weight-bearing status including physiotherapy, VTE risk assessment/prophylaxis (drug name, route, dose, when to restart, and duration), any need for elevation, neurovascular monitoring, splint application, cast change, need for special postoperative vital signs monitoring and blood tests, need for postoperative check radiographs, wound and suture removal instructions, and clinic follow-up plan. This list is not exhaustive and should be tailored to the individual patient/procedure.

Our study had certain clearly identified strengths. The audit standards were modified to suit an orthopedic trauma setting. We carried out data collection prospectively over a consecutive period to try and minimize operation note selection bias. There was consensus agreement by at least two team members for each parameter assessed as we anticipated some cases of apparent lack of clarity. We also demonstrated via our audit that simple measures like educational activities, demonstrations at clinical governance meetings, and the use of reminders can help clinicians improve operation note documentation. We also ran this audit over a six-month period to allow enough time for the implementation of the recommendations in order to achieve positive change.

Our audit study had some limitations. We could have done better in the areas of tourniquet time and EBL documentation, but this also highlights some of the challenges with effecting change via clinical audits. Utilization of a uniform EPR where almost all patient records will be on a unified, easily accessible, fast, and user-friendly system could lead to improved documentation for electronic operation notes. We found this to be a challenge that some surgeons faced when completing medical records. Evidence in the literature has shown that patient information technology systems when duplicated, slow, or complex can lead to loss of useful clinician time for medical record documentation [15,16].

## Conclusions

This closed-loop audit has demonstrated that using operation note templates (preferably typed) can improve compliance with appropriate documentation in orthopedic trauma operation notes. Active involvement of surgeons at all levels via collaborative discussion and educational interventions can lead to positive change as demonstrated in our audit. Nevertheless, a typed operation note proforma alone might not provide all the solutions unless surgeons consciously complete all relevant aspects of the proforma. Some more senior surgeons might prefer to use handwritten notes probably due to long-standing habits. This will not be a major issue if all notes are legible and properly completed. However, this perfect scenario is not usually obtainable in real practice.

We will recommend that orthopedic trauma units apply similar non-rigid templates that can be tailored to suit different categories of trauma surgery. We also plan on including anticipated blood loss during the theater briefing before surgery and documenting EBL during the WHO safety checklist sign-out as part of a planned quality improvement project. This will be part of the continuous improvement in patient care delivery even beyond the realms of a closed-loop audit. A further audit of orthopedic operation notes will be performed in one year to ensure that the current gains are sustained.
